# Research on the predicting power of the revised Tokuhashi system: how much time can surgery give to patients with short life expectancy?

**DOI:** 10.1007/s10147-019-01612-w

**Published:** 2020-01-28

**Authors:** Tamás Mezei, Anna Horváth, Péter Pollner, Gábor Czigléczki, Péter Banczerowski

**Affiliations:** 1 Department of Neurosurgery, Semmelweis University, 57 Amerikai Rd, Budapest, 1145 Hungary; 2 National Institute of Clinical Neurosciences, 57 Amerikai Rd, Budapest, 1145 Hungary; 3 3rd Department of Internal Medicine, Semmelweis University, 4 Kútvölgyi Rd, Budapest, 1125 Hungary; 4 MTA-ELTE Statistical and Biological Physics Research Group, 1/a. Pázmány Péter S., Budapest, 1117 Hungary; 5 Health Services Management Training Center, Semmelweis University, 2 Kútvölgyi Rd, Budapest, 1125 Hungary

**Keywords:** Vertebral/epidural spinal metastasis, Prognosis predicting scoring systems, Revised tokuhashi system, Surgical treatment

## Abstract

**Object:**

The primary treatment option for symptomatic metastatic spinal tumors is surgery. Prognostic systems are designed to assist in the establishment of the indication and the choice of surgical methodology. The best-known prognostic system is the revised Tokuhashi system, which has a predictive ability of about 60%. In our study, we are attempting to find the reason for its poor predictive ability, despite its proper separation ability.

**Methods:**

We have designed a one-center-based retrospective clinical trial, by which we would like to test the feasibility and the inaccuracy of the revised Tokuhashi system. In our database, there are 329 patients who underwent surgery. Statistical analysis was performed.

**Results:**

A significant increase in survival time was observed in the ‘conservative’ category. Earlier studies reported OS 0.15 at the 180-day control time, in contrast with our 0.38 OS value. The literature suggested supportive care for this category, but in our population, every patient underwent surgery. Our population passes the 0.15 OS value on day 475. We propose an adjustment of the Tokuhashi category scores. We observed significant success in resolving pain. Motor functions were improved or stabilized compared to changes in vegetative dysfunction.

**Conclusion:**

According to our results, the Tokuhashi scoring system makes very conservative predictions and prefers non-surgical palliative or supportive care. Surgical treatment increases the life expectancy of patients in poor condition. We propose modifying the therapeutic options of the revised Tokuhashi system, taking into consideration modern spine surgery techniques.

## Introduction

Metastatic epidural spinal bone tumors are major health problems nowadays, besides being an economic burden [1]. The ever-expanding oncological treatment opportunities extend the lifetime of the patients, and, consequently they affect the incidence of secondary lesions [2].

Since the publication of Patchell et al. [3], it has been accepted in literature and in practice that the primary treatment option for metastatic spine tumor is surgery (supplemented by radiotherapy in accordance with the radiosensitivity of the primary alteration) [4]. The exact surgical methodology (posterior stabilization with or without decompression, debulking, partial or en bloc spondylectomy etc.) depends on the general conditions of the individual [5, 6]. There are patients who could be treated only with non-surgical, palliative or supportive methods (oedema control, prevention of further pathological fractures, etc.) [7].

However, therapeutic decision-making is highly complicated (setting up the indication of the surgery and choosing the exact degree of invasiveness being the most difficult tasks), we have to consider many aspects [8]. Modern medicine requires the implementation of personalized solutions, in which the prognostic predicting scoring systems could help us. The “revised Tokuhashi system” (rTS) [9] perhaps is the most well-known system worldwide, however, the publications discussing its usability are also highly controversial, reporting a total predictive ability about 60% [10–12]. A highly beneficial feature of the system that besides predicting their life expectancy, it also offers therapeutic options for the patients.

Our aim was to propose new scoring thresholds in the widely used rTS, as new surgical techniques could be used to successfully treat previously inoperable patients.

## Methods

Medical database at the Department of Neurosurgery, Semmelweis University, Budapest was collected to identify all patients who underwent surgical intervention because of vertebral metastases between December 2007 and September 2015. It contains data on 382 operations performed on 337 patients, extended with the survival data of 329 patients. Only patients over the age of 18 with operated metastasis were included. Excisional surgical methods mean that the whole tumor of the vertebral body was resected (en bloc spondylectomy), and posterior stabilization was made; palliative techniques include posterior stabilization with or without posterior decompression (laminectomy) and partial tumor removal.

We have collected information about each patient, including demographic data, detailed data on the disease and the results of the surgery (see details in Table 1).

**Table 1:** Records in the database (all data are at the time of the surgical intervention)

Demographic data
Sex
Male
Female
Age
18–88 years
Description of the disease
Performance status (measured via Karnofsky Performance Scale)
KPS 10–40%
KPS 50–70%
KPS 80–100%
Main clinical symptom
Pain
Motorial deterioration
Sensorial deterioration
Vegetative deterioration
Swallowing difficulty
The combination of the above
Incidental diagnosis (radiological)
Frankel grade
A
B
C
D
E
Affected vertebral levels
C, Th, L, S
The number of the metastases (1, 2, greater or equal than 3)
Extraspinal bony metastasis
Number of the affected bone(s) (1, 2, greater or equal than 3)
Metastasis in the internal organs
The affected organ (e.g., lung, liver…)
The number of the metastasis) (1, 2, greater or equal than 3)
The operability of the metastases (removeable, unremoveable)
Primary tumor site (based on the scoring method of the revised Tokuhashi score)
0 point: lung, osteosarcoma, chondrosarcoma, stomach, bladder, esophagus, pancreas, angiosarcoma, melanoma, mesothelioma, neuroendocrine carcinoma
1 point: liver, gallbladder, unidentified
2 points: others, germ cell tumors, other epithelian carcinomas (e.g., tonsils, larynx, …), hematological malignancies, parotis
3 points: kidney, uterus, cervix, ovarium
4 points: colon, rectum
5 points: thryoid, breast, prostate, carcinoid tumor, osteoblastoma, chondroma, hemangioma
Other co-morbidities
E.g., hypertension, ischaemic hearth disease, diabetes, chronic obstructive pulmonary disease, …
Description of the hospital stay
Date of the operation
Surgery description
Excisional or radical surgical therapy
En block spondylectomy and stabilization
Partial spondylectomy and stabilization
Palliative surgical therapy
Posterior decompression and stabilization
Laminectomy or posterior decompression only
Stabilization only
Intra- and postoperative events/complications
Blood loss and required amount of transfused blood
Need for postoperative intensive care unit
The length of the hospital stay (days)
The result of the surgery
Overall, there were improvement about any of the main symptoms or not? (yes or no)
Pain
Paresis
Vegetative dysfunction

Most of these factors are part of the prognosis predictions and the therapeutic option recommendation system discussed in this study. Tokuhashi et al. published their scoring system in 1989 [13] and, after several retro- and prospective analyses, they published the revised version in 2005 (Table 2) [9]. Other systems have been proposed as well, however, the above version has had the greatest effect on the neurosurgeon society. We scored our patients according to the version published in 2005, and our use of the term ‘rTS score’ is grounded on the 2005 version.

**Table 2:** The revised Tokuhashi score

Predictive factors	Point(s)
General condition (KPS)	
Poor (KPS 10–40%)	0
Moderate (KPS 50–70%)	1
Good (KPS 80–100%)	2
No. of extraspinal bone foci	
≤ 3	0
1–2	1
0	2
No. of metastasis in the vertebral body	
≤ 3	0
2	1
1	2
Metastasis to the major internal organs	
Non-removable	0
Removable	1
No metastasis	2
Primary site of the cancer	
Lung, osteosarcoma, stomach, bladder, esophagus, pancreas	0
Liver, gallbladder, unidentified	1
Others	2
Kidney, uterus	3
Rectum	4
Thyroid, breast, prostate, arcinoid	5
Palsy	
Frankel A,B (complete)	0
Frankel C,D (incomplete)	1
Frankel E (none)	2
Prognostic categories	Interpretation
0–8 points	85% lives < 6 months ≥ conservative treatment or palliative surgery
9–11 points	73% lives > 6 months (and 30% > 1 year) ≥ palliative surgery or (exceptionally) excisional surgery
12–15 points	95% lives > 1 year ≥ excisional surgery

### Statistical examination

Descriptive statistics were used to describe the cohort of patients. We employed Fisher exact tests to identify significant correlations between covariates of interest and categorical outcomes, and we used the Kaplan–Meier formula and the log-rank test for the survival analysis (survival times are calculated from the day of the operations). To compare the success rates, we used a binomial-test. Results with *p* values less than 0.05 were considered statistically significant. All of our statistical analyses were made by R software (R Foundation for Statistical Computing, Vienna, Austria).

The research was conducted in the spirit of the Declaration of Helsinki, with the approval of the Institute’s Ethical Committee.

## Objective and previous results

The population under study has the following general properties: the sex ratio is balanced, with 199 (59.1%) males and 138 (40.9%) females in it. The typical member is from the elderly generation, as the mean age is 63, but the full age range varies between the minimum of 18 years and the maximum of 88 years. The median OS, that is, the amount of time when 50% of the patients have died is 222 days (CI 95% 175–274). OS was calculated using the Kaplan–Meier (KM) formula. Here we review the general properties of the population from the original study of the rTS [9], in which there were 154 male patients and 92 female patients. Their mean age was 56.5 years (range 15–85 years). The site of lesion is as follows: 55 cervical patients, 142 thoracic patients, and 99 lumbosacral patients. The primary site of the cancer was as follows: lung 48, breast 26, kidney 24, liver 15, prostate 15, rectum 10, stomach 10, thyroid 7, uterus 6, colon 5, stomach 5, osteosarcoma 4, unidentified 34, others 37.

In our first report, we tested the factors that have the most significant impact on survival [14]. Later, we published about the efficiency and prediction accuracy of the four best-known scoring systems used with metastatic spinal tumor patients. The systems are able to separate the patients according to their overall survival periods. However, the rTS showed 60.5%, Tomita 28.8%, modified Bauer 29.5% and the van der Linden 48.6% precision about the prediction of the real survival periods [15].

In this article, we present a plausible explanation of why the rTS has low precision despite of its statistically validated separation feasibility.

## Results

### The predicting power of revised Tokuhashi system categories

The rTS establishes three prognostic categories. We tested each of them for survival prediction: are the prognoses of survival rates in concert with the findings in the population of the data set of our study? (Nomenclature may be somewhat confusing, but in this case, the conservative, palliative and excisional appellations refer to prognostic categories, not treatment options.)

The rTS-predicted survival rate for the “conservative” category (Table 3, OS 0.15 at 180 days) is outside of the 95% confidence interval of the 180 days survival rate of our data (OS 0.38, CI 95% 0.31–0.47). However, it is worthy of note that in the original Tokuhashi study, the prognostic values were calculated with patients who only received conservative treatment and did not undergo surgery. In our database, we have no data of patients who received this type of therapy only.

**Table 3:** Survival rates at typical time points for rTokuhashi prognostic values, “conservative category”

Time (days)	OS	Standard error	Lower 95.00% CI	Upper 95.00% CI	Literature
30	0.8623	0.0293	0.8067	0.922	
60	0.7319	0.0377	0.6616	0.81	
90	0.6449	0.0407	0.5698	0.73	
**180**	**0.38**	**0.0416**	**0.3066**	**0.471**	**0.15 ≥ We have higher survival**
365	0.193	0.035	0.1352	0.275	
1095	0.0505	0.0209	0.0224	0.114	
1825	0.0337	0.0196	0.0108	0.105	

Bold line shows the survival time period what is belong to the rTokuhashi's 0-8 point(s) category

In the “palliative” category (Table 4) the first rTS-predicted survival rate (OS 0.73 at 180 days) is within the 95% confidence interval of the 180 days survival rate of our data (OS 0.65, CI 95% 0.58–0.74). The second predicted survival rate (OS 0.30 at 365 days) is lower than the 365 days survival rate in our population (OS 0.54, CI 95% 0.46–0.64). We conclude that surgery provides longer OS for patients who have survived the first critical half-year period.

**Table 4:** Survival rates at typical time points for rTokuhashi prognostic values, “pallative category”

Time (days)	OS	Standard error	Lower 95.00% CI	Upper 95.00% CI	Literature
30	0.969	0.0155	0.939	0.999	
60	0.874	0.0294	0.818	0.934	
90	0.803	0.0353	0.737	0.875	
**180**	**0.653**	**0.0423**	**0.575**	**0.741**	**0.73 = > Our population is not different**
**365**	**0.544**	**0.0447**	**0.463**	**0.639**	**0.30 = > We have higher survival**
1095	0.281	0.0453	0.205	0.386	
1825	0.24	0.0474	0.163	0.353	

Bold lines show the survival time period what are belong to the rTokuhashi's 9-11 points category

We compared the effect of surgery types on the survival rates of the patients in the palliative rTS score category. We have defined two subgroups of patients: the first subgroup received the suggested surgery, the other one received more radical surgery. We found seemingly large, but no significant difference (*p* = 0.08) in survival between the two subgroups.

Lastly, for the “excisional” category (Table 5), the predicted survival rate (OS 0.95 at 365 days) is higher than the upper limit of the 95% confidence interval of the 365 days survival rate of our data (OS 0.54 CI 95% 0.42–0.69). We expect a considerable effect of the medical cultural behavior and genetic differences between the population in our study and in those of the population examined in the study of rTS [9]. Differences in living conditions can account for the observed difference for the longest living group.

**Table 5:** Survival rates at typical time points for rTokuhashi prognostic values, “excisional categories”

Time (days)	OS	Standard error	Lower 95.00% CI	Upper 95.00% CI	Literature
30	0.966	0.0236	0.921	1	
60	0.932	0.0327	0.87	0.999	
90	0.864	0.0446	0.781	0.956	
180	0.72	0.0596	0.612	0.847	
**365**	**0.541**	**0.0686**	**0.422**	**0.693**	**0.95 = > We have lower survival**
1095	0.368	0.0755	0.246	0.55	
1825	0.26	0.0749	0.148	0.457	

Bold line shows the survival time period what is belong to the rTokuhashi's 12–15 points category

We have compared the survival of two subgroups of patients who were recommended for excisional surgery based on their rTS score. The first subgroup had the suggested surgery, the other one had some other type of surgery. We found no significant difference (*p* = 0.73) in survival rates between the two subgroups.

### Analysis of the ‘conservative’ category

In this subsection, we focus on the ‘conservative’ category group. This category contains 132 patients. For finding possible causes of longer OS than predicted, we defined two subgroups of patients: the first subgroup has an OS rate as predicted by the rTS, and the second one is the group with longer-living patients. To classify patients into these subgroups consistently, we distributed all patients with a Monte Carlo simulation between the two groups, and accepted the permutation that had the lowest variance in OS. The first group with the predicted OS time consists of 84 patients, and 48 of them are classified as longer-living patients. In our population, the OS value decreased to 0.15 on day 475 (CI 95% 359–796) (contrary to the 180th day in the literature).

#### Proposing a new maximum score for conservative treatment

**Fig. 1:**
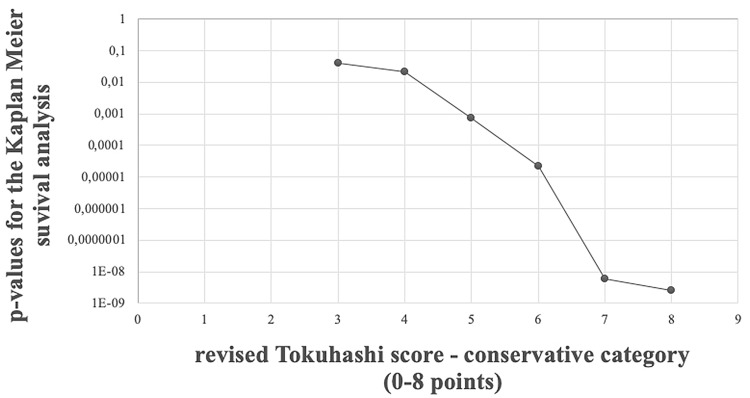
*p* values of the log-rank test for the difference between Kaplan–Meier survival of the conservative group, when the “score limit” is the upper revised Tokuhashi score value for the conservative group

**Fig. 2:**
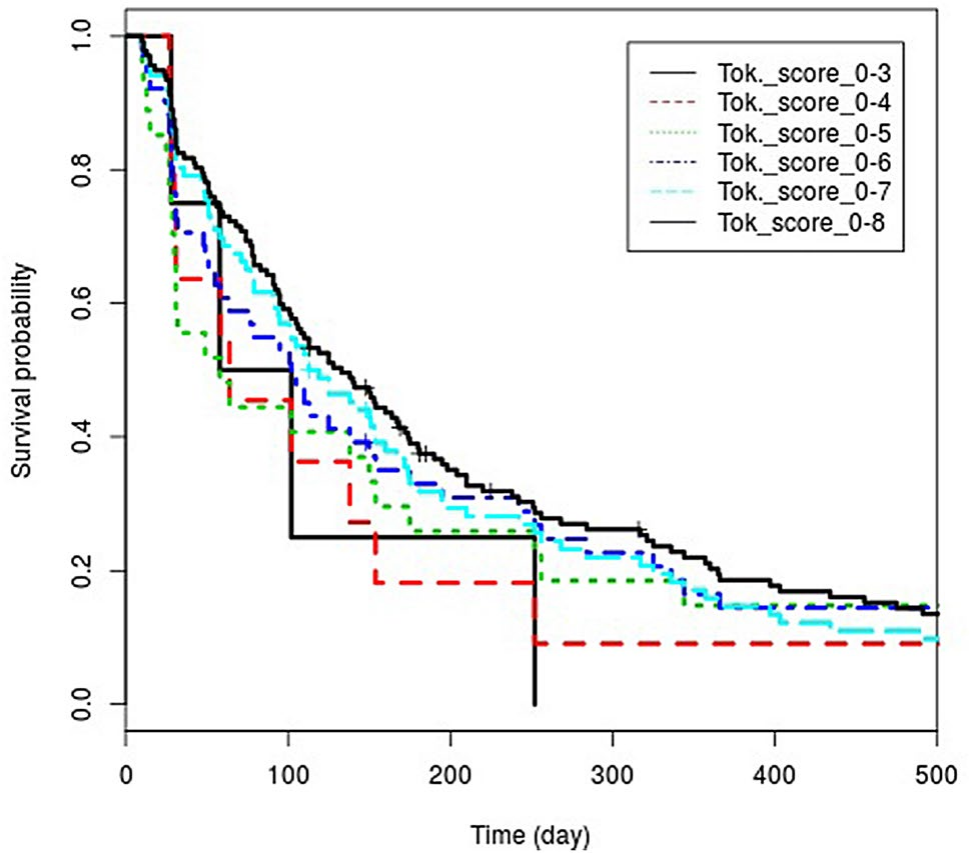
Kaplan–Meier curves of the conservative category

Another way of grouping the conservative category into two subgroups is by utilizing the rTS score. The first group consists of patients with low scores, and the other one consists of patients with high scores. Patients in the group with low scores are forming the new, proposed conservative category. Patients with high scores are classified into the palliative category. We have investigated the OS difference between the two rTS score categories (conservative with changed upper score boundary and palliative with changed lower score boundary) when the boundary score was altered. We can observe, that the OS difference (measured by the *p* value of the log-rank test) does not change, when the cutoff score between the conservative and palliative category changes from 8 to 7, but at the cutoff score 6 we see rapid change in the significance level. (Fig. 1.). For general reference, we provide KM curves for all individual scores as well in Fig. 2.

Based on these results, we propose a new upper limit for the conservative treatment. Instead of the earlier recommendation of rTS, which is a score of 8, we recommend using the value of 6.

#### Dominant factor in the group with higher scores

We have investigated the confusion matrix of all prognostic factors in the subgroup with higher scores of the conservative category**.** We found that the primary site of the tumor is the most important factor in the survival. In the group with higher rTS scores (belonging to the conservative category) we found significantly more hematologic malignancy (*p* = 0.020, OR 4.05, 95% CI 1.17–16.17) and less lung cancer cases (*p* = 0.008, OR 0.37, 95% CI 0.16–0.81).

#### Quality of life factors

In this subsection, we present results about the differences in factors affecting the quality of life, between the high and low rTS-score groups within the conservative category. As we have seen at the definition of the subgroups, patients with higher scores live significantly longer. Maybe a very important question must be answered for the patients: is the gained surviving time a real gain with better living conditions, or is it simply an elongation of the therapy? We have investigated the improvement in motor movements and the experienced pain, as these are the most significant deteriorating factors affecting the quality of life that we have data on.

The results can be seen in Table 6. We see no significant difference between the groups in terms of factors affecting the quality of life: the ratio of patients with these factors are not different (pain *p* = 0.533, OR 1.35, 95% CI 0.54–3.55 and motorial dysfunction *p* = 0.587, OR 1.30, 95% CI 0.60–2.82). Before concluding that extending a patient’s life would mean elongating their suffering, we note that, with such population size, the improvement cannot be measured.

**Table 6:** Solution of symptoms in the two subgroups of the conservative category

	Symptoms in total		Success rate (%)	Pain		Success rate (%)	Motorial dysfunction		Success rate (%)
	Improve (case no.)	Decrease (case no.)		Improve (case no.)	Decrease (case no.)		Improve (case no.)	Decrease (case no.)	
Longer-survival subgroup (*n* = 48)	47	1	98	42	6	88	22	26	45
Shorter-survival subgroup (*n* = 84)	83	1	99	80	4	95	44	40	52

From the 132 patients of the conservative treatment group, 130 achieved improvement in the experienced pain, which is a significantly good prediction for the surgical intervention. The only two cases with no pain improvement do not allow for statistical hypothesis tests to establish differences between any two subgroups in this population.

The third quality of life factor is vegetative dysfunction. We can say that the surgical treatment of the existing urine or stool incontinence is the most difficult one, compared to paresis (*p* = 0.001, OR 5.01, CI 95%1.86–13.97) and pain (*p* < 1.e5, OR 10.64, CI 95%3.81–31.35) both of the other two can be cured more effectively.

## Discussion

As an oncological disease progresses, the chance of developing metastatic bone diseases (including metastatic spine tumors) increases, which means poor prognosis for the patients [2, 16].

The highly malignant glioblastoma multiforme, which is still an unresolved oncological problem, has a median survival time of 14.6 months [17]. However, our metastatic spinal tumor population has a lower value, with a median OS time of 7.5 months. The above data are identical to what has been published in the literature. Rades et al. [18] found a 2-, 4- and 11-months median OS time for their three subgroups of patients who underwent radiotherapy without surgery, da Silva et al. [19] examined a population with secondary lesions caused by lung carcinoma, and described a median OS value between 8 and 12 months.

Prognosis-predicting scoring systems establish the categorization of patients based on their survival time, which can help doctors choose the optimal type of treatment [20, 21]. This separation ability is also validated by the literature [22], nonetheless, more and more articles are being produced about the feasibility of the systems. Our previous study showed an average predictive capability of 60% for the rTS, which is also consistent with literature data. Zoccali et al. [23] reported an average 63% predictive value for rTS. In their results, it can be seen that the prediction ability of the system is reduced at the patients with a life expectancy shorter than 1 year (more than 12 months: 77.21%, 6–12 months: 55.32% and less than 6 months: 64.10%).

The main question of our study is what the cause of the inaccurate prediction of the system could be. In our conservative category population, a significant increase in survival time was observed, the OS value decreased to 0.15 on day 475, contrary to the 180th day in the literature, which means 295 days of lifetime elongation. The reason for the increase in survival time was found in differences of the treatment options, namely, while all of our patients underwent surgery, literature suggests only supportive (or non-surgical palliative-) therapy for this category. Pelegrini et al. [24] also criticized the recommendation of treatment methods of the rTS, the advantages of surgical treatment were emphasized against conservative options. Oliveira et al. [25] also reported that the therapy cannot be based on the recommendation of the rTS.

Based on our results, we tried to change the conservative category point limits where there was a significant difference in therapy. Instead of the 0–8 points found in the literature, we would change the conservative category points to 0–6.

It could be seen again; the primary tumor type is one of the most important prognostic factors influencing the outcome of the disease. The subgroup of the conservative category examined in detail, which contained patients who did not survive the predicted 6-month period, contained significantly more lung cancer patients. The study prepared by Bollen et. al [26] also highlights the above statement (the primary tumor and the performance status are the most important factors affecting survival). Even more prognosis-predicting scoring systems appear in the literature which focus on metastases caused by one type of primary tumor (so we can get more precise prediction based on more specific information, e.g., histological categorization, molecular subtypes, specific targeted treatment options, etc.). Uei et al. [27] designed a new system for estimating the outcome of spinal metastasis in patients with primary lung cancer, which also takes into account the possibility of tumor antigens and molecular target therapy.

The spread of minimally invasive spine surgery techniques also allows for surgical intervention for patients with poor general condition due to lower surgical load. For example, spinal stabilization procedures could be performed with less blood loss and surgical strain [28, 29], or a metastatic epidural tissue mass causing metastatic epidural spinal cord compression can be removed with minimal access hemi-semi laminectomy [30].

By conserving neurological functions, we can achieve significant increases in the patients’ quality of life and survival time. Several studies have shown that surgery has a good effect on the quality of life of patients [31, 32], de Ruiter et al. [33] reported that invasive open surgery techniques do not negatively affect patient outcomes. By preventing neurological functions, side effects (such as thrombosis, decubitus, infections, etc.) can be avoided. The extension of the survival time of our own population is also attributed to the absence of the above ‘side effects’. To sum up, in determining the therapeutic options for the subcategories of the prognosis-predicting scoring systems, we must take into account the open-access opportunities offered by minimal access surgery techniques, as described by Rao et al. [34].

## Conclusion

The treatment of epidural spinal metastases is a major challenge from both oncological and surgical aspects. In doubtful cases, prognosis-predicting systems could help, however, but we would like to emphasize that they only serve as guidance and are of secondary importance compared to a doctor’s years or decades of medical experience.

According to our results, rTS draws a too strict boundary between surgical and other, non-surgical palliative and/or supportive therapeutic options, as it may be worthwhile to also provide surgery for patients who are in poor condition based on the scoring systems. Surgical treatment is unquestionably important for the treatment of pain and may also lead to further improvement in the quality of life of a patient through the improvement/preservation of neurological functions, which may make patients eligible for further oncological treatments. Based on the above, we recommend the revision of the therapeutic recommendation section of the rTS system, also considering the modern spinal surgery techniques.

## Abbreviations

CI: Confidence interval
KM: Kaplan–Meier
OR: Odds ratio
OS: Overall survival
*p*: Probability
rTS: Revised Tokuhashi system
